# Genomic characterization of SARS-CoV-2 from an indigenous reserve in Mato Grosso do Sul, Brazil

**DOI:** 10.3389/fpubh.2023.1195779

**Published:** 2023-10-26

**Authors:** Laís Albuquerque de Oliveira, Izabela Mauricio de Rezende, Vinicius João Navarini, Silvana Beutinger Marchioro, Alex José Leite Torres, Julio Croda, Mariana Garcia Croda, Crhistinne Cavalheiro Maymone Gonçalves, Joilson Xavier, Emerson de Castro, Mauricio Lima, Felipe Iani, Talita Adelino, Flávia Aburjaile, Luiz Henrique Ferraz Demarchi, Deborah Ledesma Taira, Marina Castilhos Souza Umaki Zardin, Vagner Fonseca, Marta Giovanetti, Jason Andrews, Luiz Carlos Junior Alcantara, Simone Simionatto

**Affiliations:** ^1^Health Sciences Research Laboratory, Federal University of Grande Dourados, Dourados, Mato Grosso do Sul, Brazil; ^2^Stanford Pandemic Preparedness Hub, Department of Medicine, Division of Infectious Diseases and Geographic Medicine, Stanford University School of Medicine, Stanford, CA, United States; ^3^Laboratory of Immunology and Molecular Biology, Institute of Health Sciences, Federal University of Bahia, Salvador, Bahia, Brazil; ^4^Oswaldo Cruz Foundation, Campo Grande, Mato Grosso do Sul, Brazil; ^5^Faculdade de Medicina (FAMED), Universidade Federal do Mato Grosso do Sul, Campo Grande, Mato Grosso do Sul, Brazil; ^6^School of Medicine, Federal University of Mato Grosso do Sul, Campo Grande, Mato Grosso do Sul, Brazil; ^7^State Secretariat of Health of Mato Grosso do Sul, Campo Grande, Mato Grosso do Sul, Brazil; ^8^Federal University of Minas Gerais, Belo Horizonte, Minas Gerais, Brazil; ^9^Ezequiel Dias Foundation (FUNED), Belo Horizonte, Minas Gerais, Brazil; ^10^Preventive Veterinary Medicine Departament, Veterinary School, Universidade Federal de Minas Gerais, Belo Horizonte, Brazil; ^11^Central Public Health Laboratory (Lacen), Campo Grande, Mato Grosso do Sul, Brazil; ^12^Pan American Health Organization - PAHO, Brasília, Distrito Federal, Brazil; ^13^Rene Rachou, Fundação Oswaldo Cruz, Belo Horizonte, Minas Gerais, Brazil; ^14^Sciences and Technologies for Sustainable Development and One Health, Università Campus Bio-Medico di Roma, Rome, Italy; ^15^Climate-Amplified Diseases and Epidemics (CLIMADE) Rio de Janeiro, Rio de Janeiro, Brazil

**Keywords:** SARS-CoV-2, COVID-19, indigenous population, VoI, VOC, pandemic

## Abstract

**Background:**

The COVID-19 pandemic had a major impact on indigenous populations. Understanding the viral dynamics within this population is essential to create targeted protection measures.

**Methods:**

A total of 204 SARS-CoV-2 positive samples collected between May 2020 and November 2021 from an indigenous area in Mato Grosso do Sul (MS), Midwestern Brazil, were screened. Samples were submitted to whole genome sequencing using the Nanopore sequencing platform. Clinical, demographic, and phylogenetic data were analyzed.

**Results:**

We found the co-circulation of six main SARS-CoV-2 lineages in the indigenous population, with the Zeta lineage being the most prevalent (27.66%), followed by B.1.1 (an ancestral strain) (20.21%), Gamma (14.36%) and Delta (13.83%). Other lineages represent 45.74% of the total. Our phylogenetic reconstruction indicates that multiple introduction events of different SARS-CoV-2 lineages occurred in the indigenous villages in MS. The estimated indigenous population mortality rate was 1.47%. Regarding the ethnicity of our cohort, 64.82% belong to the Guarani ethnicity, while 33.16% belong to the Terena ethnicity, with a slightly higher prevalence of males (53.43%) among females. Other ethnicities represent 2.01%. We also observed that almost all patients (89.55%) presented signs and symptoms related to COVID-19, being the most prevalent cough, fever, sore throat, and headache.

**Discussion:**

Our results revealed that multiple independent SARS-CoV-2 introduction events had occurred through time, probably due to indigenous mobility, since the villages studied here are close to urban areas in MS. The mortality rate was slightly below of the estimation for the state in the period studied, which we believe could be related to the small number of samples evaluated, the underreporting of cases and deaths among this population, and the inconsistency of secondary data available for this study.

**Conclusion:**

In this study, we showed the circulation of multiple SARS-CoV-2 variants in this population, which should be isolated and protected as they belong to the most fragile group due to their socioeconomic and cultural disparities. We reinforce the need for constant genomic surveillance to monitor and prevent the spread of new emerging viruses and to better understand the viral dynamics in these populations, making it possible to direct specific actions.

## 1. Introduction

The Coronaviruses disease 2019 (COVID-19) pandemic, caused by a new coronavirus named severe acute respiratory syndrome coronavirus 2 (SARS-CoV-2), has spread globally with unimaginable proportions, reaching populations worldwide and causing thousands of deaths (1). Among the populations affected by the pandemic, indigenous peoples were substantially impacted, with irreparable human and cultural losses. Several factors, such as cultural, social and biological aspects, have a significant impact on the transmissibility and occurrence of infectious diseases in these populations, leaving them in a situation of greater fragility and vulnerability (2–4).

The impact of SARS-CoV-2 on global public health and economies has been profound (5). To contain the virus spread globally and to reduce the epidemic to growth, public health interventions and non-pharmaceutical measurements were adopted. Social distancing, borders and travel restrictions, lockdowns, mask wearing, and contact tracing were some of the interventions that have shown effectiveness in mitigating the spread of COVID-19 (6, 7). Shortly in the pandemic, in December 2020, the U.S. Food and Drug Administration (FDA) issued the first emergency use authorization for use of the Pfizer-BioNTech COVID-19 vaccine in persons aged 16 years and older for the prevention of COVID-19 (8). In Brazil, the COVID-19 vaccination calendar starter in January 2021, including indigenous population over 18 years old among the priority group (9). However, when compared to the general population, the indigenous population achieved lower vaccination coverage (4).

Since January 10, 2020, up to December 2022, Brazil has confirmed 36,960,888 COVID-19 cases and 697.894 confirmed deaths (9). Among the currently available public SARS-CoV-2 genome sequences, data on sequencing of COVID-19 cases from indigenous people were not found in the literature, evidencing the paucity of data and reinforcing the need to expand genomic surveillance in groups of ethnic minorities, underserved and isolated.

**Figure 1:**
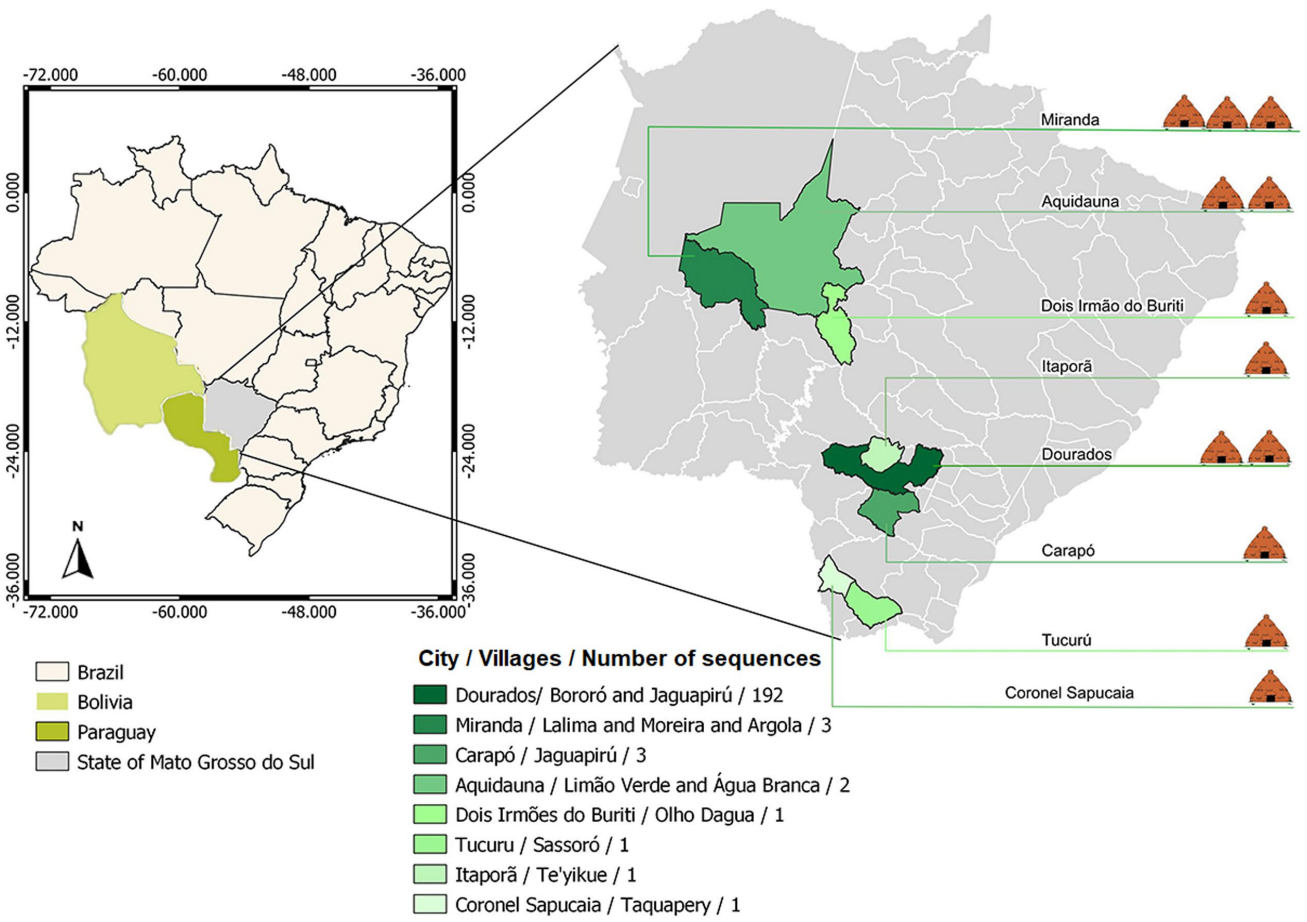
Geographical distribution of the villages covered by the study.

Brazil has an estimated indigenous population of 1,108,970 people (0.5% of the Brazil population – 214,300,000) living in indigenous areas, subdivided into more than 300 ethnic groups that speaks more than 274 different languages (3, 10). In Mato Grosso do Sul (MS) state, Midwestern of Brazil, this population represents approximately 3% of the state’s population. In MS, indigenous villages are located close to urban areas, where social and commercial relations with non-indigenous people could contribute to the spread of COVID-19 among indigenous peoples. About 65% of the indigenous population lives in the south of the state, where the city of Dourados is located (11). In this city, around the Dourados-Itaporã highway, is located the Dourados Indigenous region, the largest Brazilian peri-urban indigenous site, with a total area of 3,474,59 acres and a population of approximately 18,000 inhabitants, living in Bororó and Jaguapiru villages (12–14) (Figure 1).

Mato Grosso do Sul state ranked first in the number of confirmed cases of SARS-CoV-2 infections and second in the number of deaths in the indigenous population (3), when compared to other states in Brazil during the period of this study (15). The genetic characterization of viruses is essential not only for developing vaccine and antiviral protocols aiming for efficient treatments and diagnosis but also for monitoring and controlling disease outbreaks, following their evolution, and supporting decision-making (16).

The Brazilian natives have suffered heavily from the impacts caused by COVID-19 since the beginning of the pandemic. This research aimed to genomic characterized SARS-CoV-2 circulating strains in the indigenous population of Mato Grosso do Sul state, mainly in the Dourados Indigenous Reserve area, from May 2020 to November 2021.

## 2. Methods

### 2.1. Clinical and sociodemographic data

The sociodemographic and clinical data of participants in this study were extracted from data spreadsheets released by the Special Indigenous Health District (DSEI) of Mato Grosso do Sul, a decentralized management unit of the Indigenous Health Care Subsystem (SasiSUS) in Brazil. These professionals are part of the staff of the Special Indigenous Health Secretary (SESAI). Each health agent has a number of households to monitor as well as their residents. The indigenous health data collected by the agents are stored in the Information System for Indigenous Health Care (SIASI) and in the Information System of the National Immunization Program (SI-PNI), preserving the confidentially of individuals/patients. This study was approved by The National Research Ethics Committee (CONEP) with identification number 4.584.624.

### 2.2. Samples collection and molecular diagnostic assays

Clinical samples of indigenous patients with suspected SARS-CoV-2 infection and residing in indigenous areas of Mato Grosso do Sul state were collected from May 2020 to November 2021 for COVID-19 diagnosis and whole genome sequencing. The biological material was collected following the workflow already established in the Basic Indigenous Health Units (UBSI) for detecting SARS-CoV-2 using qRT-PCR (17). Viral RNA was extracted from nasopharyngeal swabs using QIAamp Viral RNA Mini KIT (QIAGEN), following manufacturer’s instructions. The COVID-19 diagnosis was performed using one of the three protocols of qRT-PCR: (1) the Allplex 2019-nCoV assay (Seegene) targeting the envelope (E), the RNA-dependent RNA polymerase (RdRp) and the nucleocapsid (N) genes; (2) SARS-CoV2 (E/RP) assay (Bio-Manguinhos/Fiocruz) targeting the E and RP gene; and (3) BioMol oneStep/COVID-19 kit (Institute of Molecular Biology of Paraná (IBMP)) targeting ORF-1ab and N gene. All protocols were performed following the manufacturers’ instructions.

### 2.3. Whole-genome sequencing

Positive samples were selected for sequencing based on the Ct value (≤25). The SARS-CoV-2 sequencing was performed using the Oxford Nanopore technology. Briefly, SuperScript IV Reverse Transcriptase kit (Invitrogen) was initially used for complementary DNA (cDNA) synthesis, following the manufacturer’s instructions. The cDNA generated was then subjected to multiplex PCR using the Q5 High-Fidelity Hot-Start DNA Polymerase (New England Biolabs) and a set of specific primers designed by the ARTIC Network for sequencing the complete SARS-CoV-2 genome (Artic Network version 3), as previously described (18).

Amplicons were purified using 1x AMPure XP beads (Beckman Coulter) and quantified on Qubit (ThermoFisher) using Qubit dsDNA HS assay kit (ThermoFisher). DNA library preparation was performed using the ligation sequencing kit LSK109 (Oxford Nanopore Technologies) and the native barcoding kit (NBD104 and NBD114, Oxford Nanopore Technologies). Sequencing libraries were loaded into an R9.4 flow cell (Oxford Nanopore Technologies). In each sequencing run, we used negative controls to prevent and check for possible contamination with less than 2% mean coverage.

### 2.4. Genome assembling and lineage analyses

Sequencing raw files were base called using Guppy v3.4.5 and barcode demultiplexing was performed using qcat. Consensus sequences were generated by *de novo* assembling using Genome Detective (19) that uses DIAMOND to identify and classify candidate viral reads in broad taxonomic units, using the viral subset of the Swissprot UniRef protein database. Candidate reads were next assigned to candidate reference sequences using NCBI blastn and aligned using AGA (Annotated Genome Aligner) and MAFFT. Final contigs and consensus sequences were available as FASTA files.

To ensure the quality of the genome sequences generated in this study and to guarantee the highest possible phylogenetic accuracy, only genomes >29,000 bp with <1% of ambiguities were considered. The genomes were submitted to Pangolin COVID-19 Lineage Assigner Tool v.3.1.142 to confirm the variant classification.^1^

### 2.5. Phylogenetic analysis

The datasets used for the phylogenetic analysis included Brazilian SARS-CoV-2 complete genomes sequences retrieved from the GISAID database.^2^ References genomes of each variant were added according to the GISAID initiative using the region-specific download source on the website. Nucleotide sequences were aligned using MAFFT (20) and submitted to IQ-TREE2 for maximum-likelihood (ML) phylogenetic analysis (21), employing the general time reversible model of nucleotide substitution (GTR + F + R4) according to Bayesian Information Criterion (BIC), inferred by ModelFinder application (22). Branch support was assessed using Ultrafast Bootstrap (UFBoot) 1,000 replicates. TreeTime (23) was used to transform this ML tree topology into a dated tree using a constant mean rate of 8.0 × 10^−4^ nucleotide substitutions per site per year, after excluding outlier sequences.

### 2.6. Data availability statement

All sequences generated and used in the present study are listed in Supplementary Table 1, along with their GISAID sequence IDs, dates of sampling, and the originating.

## 3. Results

### 3.1. Indigenous population and COVID-19 clinical data in an indigenous population

For the clinical and sociodemographic analysis, data from the 204 indigenous patients were obtained. When analyzing data related to the village, 55.67% were from Jaguapiru and 32.51% were from Bororó villages (Figure 1 and Table 1). Other communities added up to 11.82% (Table 1). Out of the participants, 64.82% belong to the Guarani ethnicity, while 33.16% belong to the Terena ethnicity. Other ethnicities represent 2.01% (Table 1).

**Table 1:** Demographic and clinical information from indigenous populations participating in this study.

Variable	*N* (206)	%
Age		
<18 years	31	15.04
18–30	52	25.24
31–40	52	25.24
41–50	32	15.53
51–60	22	10.67
>60 years	14	6.79
SARS-CoV-2 variant detected		
AY.101	13	6.31
B.1.1	38	18.44
B.1.1.28	15	7.28
B.1.1.33	15	7.28
P.1	29	14.07
P.2	52	25.24
Not detected	16	7.76
Others*	28	13.59
Gender		
Female	95	46.11
Male	111	53.88
City		
Dourados	198	96.11
Others	8	43.88
Village		
Jaguapirú	113	54.85
Bororó	66	32.03
Others	27	13.10
Ethnicity		
Guarani-Kaiowa	110	53.39
Terena	66	32.03
Others	23	11.16
Symptoms		
Light	161	78.15
Asymptomatic	27	13.10
Severe	18	8.73
Severe Respiratory syndrome (SR)		
Yes	116	56.31
No	56	27.18
Not reported	34	16.50
Severe Acute Respiratory Syndrome (SRAG)		
Yes	7	3.39
No	165	80.09
Not reported	34	16; 0.50
Comorbidities		
No	195	94.66
Yes	11	5.33
Hospitalized Patient		
No	172	83.49
Yes	9	4.36
Not reported	25	12.13
Patient with 14-day travel history		
No	159	77.18
Not reported	47	22.81
Patient reported a contact with a confirmed COVID-19 case		
Yes	112	54.36
No	20	9.70
Not reported	74	35.92
Hospitalization before symptoms onset		
No	156	75.72
Yes	5	2.42
Not reported	45	21.84
Disease outcome		
Cure	144	69.90
Death	3	1.45
Not reported	59	28.64
Influenza vaccination status		
Yes	18	7.28
No	5	2.42
Not reported	183	88.83
COVID-19 vaccination status		
Yes	22	10.67
No	9	4.36
Not reported	175	84.95

*Other variants detected at lower frequency AY.43; B.1; B.1.1.161; B.1.1.411; B.1.1.203; B.1.1.54; B.1.617.1; N.4.

We also compare gender information and found a slightly higher prevalence of males (53.43%) among females. When we stratify the information by age group, most of the indigenous participants belong to the age range between 18 and 39 years old (46.57%), followed by the ages between 40 and 60 years (31.37%). Participants under 18 years represent 14.71%, and older adult indigenous indigenous, older than 60 years, represent a total of 7.35% of the participants (Table 1). That can be a direct reflection of the community structure, that is characterized by a younger age structure due to higher birth rates and early mortality (24).

In this study, 89.55% of patients showed any sign or symptom of COVID-19 disease, and the most frequents were cough (71.66%), fever (48.49%), sore throat (47.78%), and headache (41.11%). Were also reported, but less frequently, running nose (33.89%), myalgia/arthralgia (8.91%), loss of smell (9.46%), loss of taste (9.44%), and vomit (7.78%). Other symptoms, such as diarrhea, difficulty of breathing, dyspnea, and low O_2_ saturation had a percentage of less than 5% in our population (Table 1). We analyzed the hospitalization and clinical evolution of the patients and found that 5.03% were hospitalized, 68.65% was discharged after clinical improvement and, 1.62% evolved to death (Table 1).

We also analyzed close contacts with suspected or confirmed COVID-19 cases and travel history of the reported cases (Table 1). Travel history of 14 days before the onset of symptoms was not reported, indicating that all cases probably occurred within the villages (Table 1). A total of 69.62% of the patients reported close contact with suspected cases, and 79.29% reported contact with a confirmed COVID-19 case (Table 1).

### 3.2. SARS-CoV-2 phylogenetic inferences and lineages diversity in indigenous peoples

A total of 204 positive samples for SARS-CoV-2 from the indigenous population included in this study were obtained, including samples from 3 patients that died, constituting a mortality rate of 1.47%. To understand the dynamics of SARS-CoV-2 in the Brazilian indigenous population, we analyzed clinical and sociodemographic data within phylogenetic analysis using a data set comprising available representative genomes from Brazil, including the genomes sequenced in this study.

**Figure 2:**
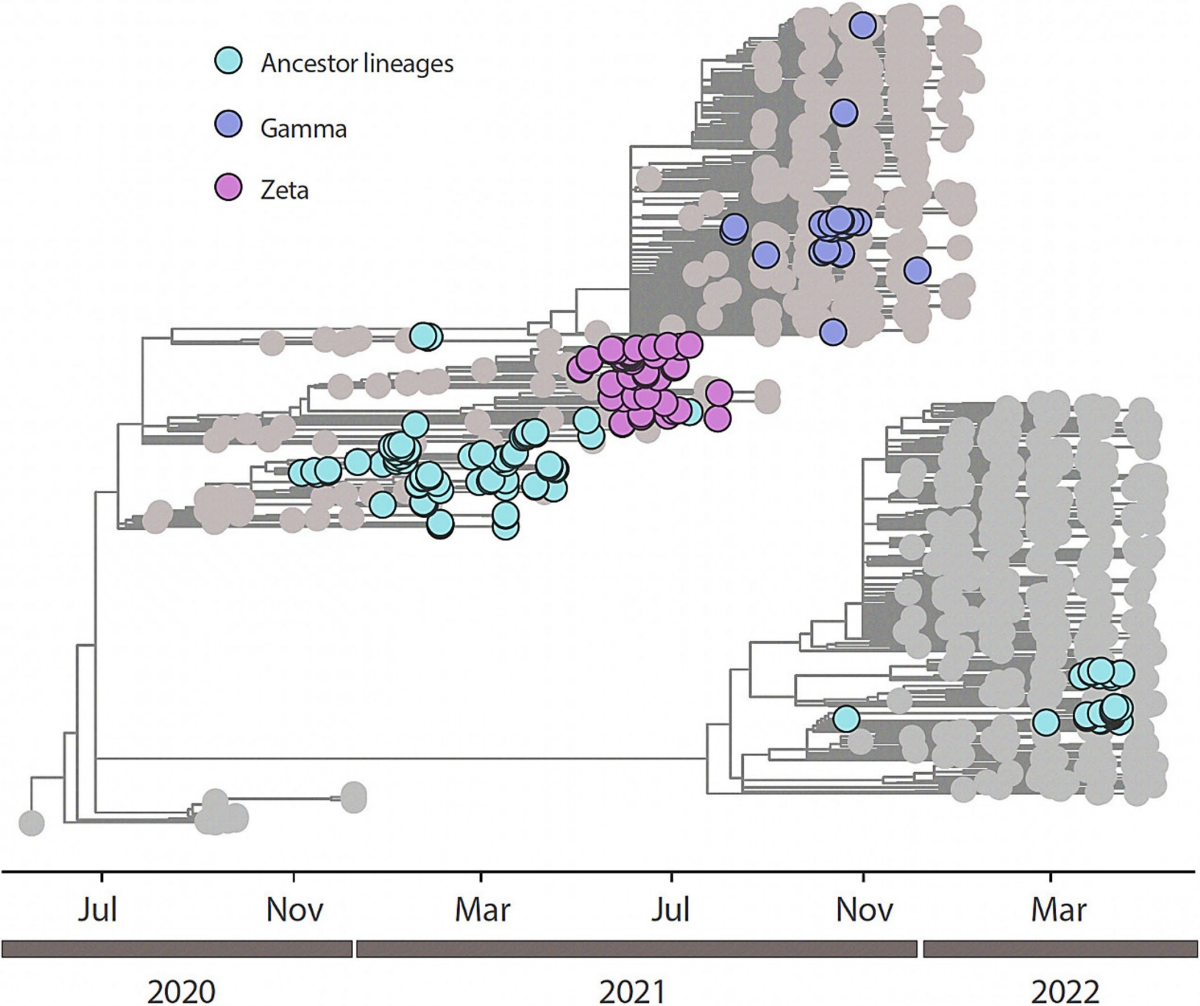
Time-resolved maximum-likelihood tree of n=188 newly SARS-CoV-2 whole genome sequences generated from this study in addition with n=846 reference strains collected at GISAID up to February 26th, 2023. Newly genomes are colored according to their lineage assignment.

**Figure 3:**
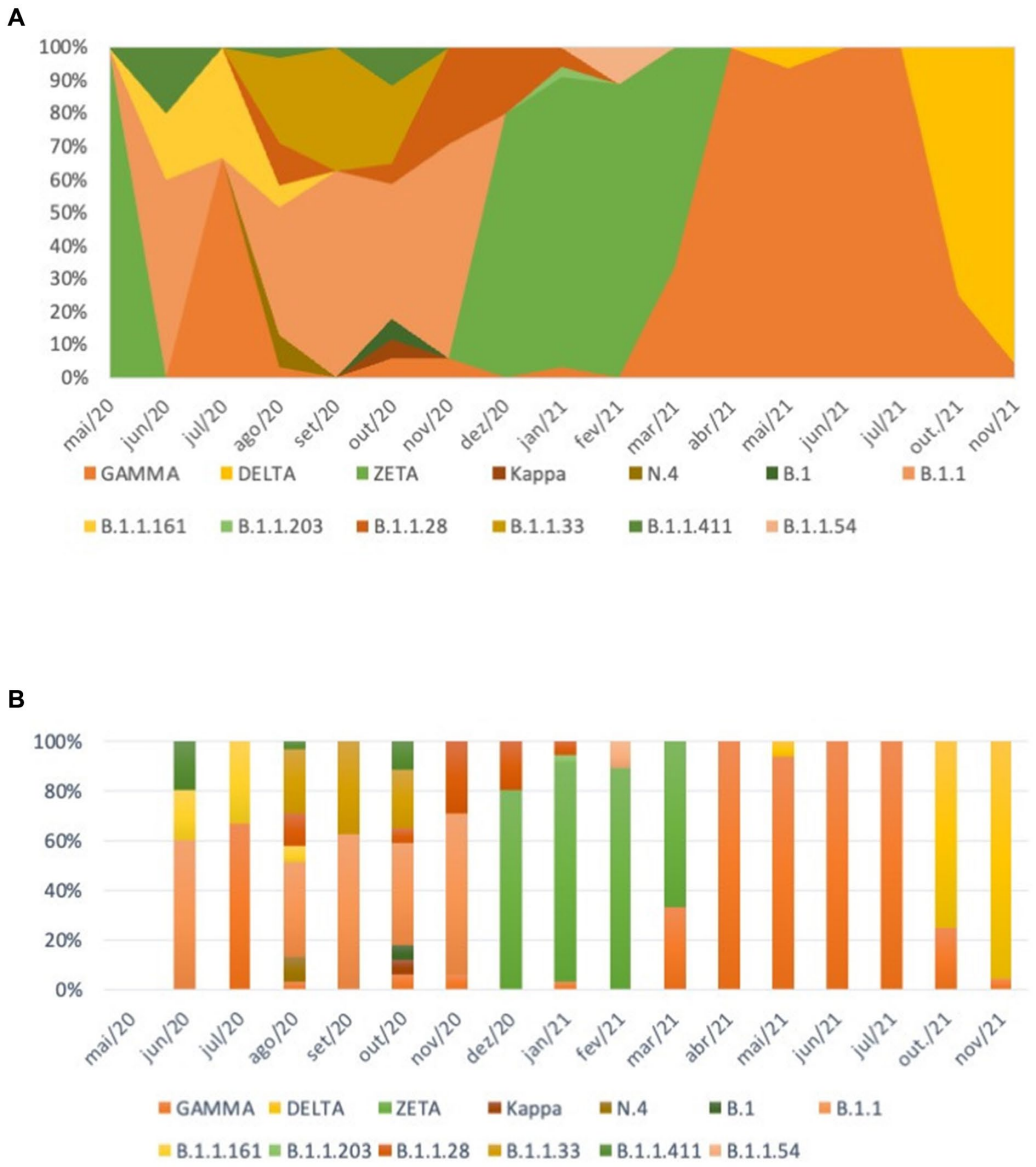
Distribution of SARS-CoV-2 variants over time. **(A)** Distribution of SARS-CoV-2 Pangolin strains according to symptom onset. **(B)** Distribution of SARS-CoV-2 strains according to the nomenclature proposed by the WHO Technical Advisory.

All the 204 samples that were qRT-PCR positive for SARS-CoV-2 had a compatible Ct value (≤25) for sequencing. We retrieved 188/204 nearly completed genomes (Figure 2). Using Pangolin (see text footnote 1), we classified the 188 sequences generated in this study into SARS-CoV-2 lineages (Supplementary Table 1). Our data shows the co-circulation of 13 distinct lineages of SARS-CoV-2 in the indigenous population from this study (Supplementary Table 2), as follows: 27.66% were classified as belonging to the Zeta (P.2), 20.21% to B.1.1, 14.36% to Gamma (P.1), 13.83% to Delta (AY101/AY43/B.1.617.2), 7.98% to B.1.1.28, 7.98% to B.1.1.33, 2.13% to B.1.1.161, 2.13% to B.1.1.411, and 1.60% to N4 lineages. Other lineages, with less representation (Kappa (B.1.617.1); B.1; B.1.1.203; and B.1.1.54) had a value of 0.53% each, of the total (Figure 3 and Table 2).

**Table 2:** SARS-CoV-2 lineages per village and ethnicity.

SARS-CoV-2 Lineages (n)	Ethnicity n (%)			Villages n (%)		
	Guarani	Terena	Others	Jaguapirú	Bororó	Others
Zeta (52)	41 (78.85)	10 (19.23)	1 (1.92)	32 (61.54)	15 (28.85)	5 (9.62)
B.1.1 (38)	25 (65.79)	12 (31.58)	1 (2.63)	16 (42.11)	17 (44.74)	5 (13.16)
Gamma (27)	13 (48.15)	11 (40.74)	3 (11.11)	16 (59.26)	1 (3.70)	10 (37.04)
Delta (26)	14 (53.85)	12 (46.15)	0 (0)	17 (65.38)	9 (34.62)	0 (0)
B.1.1.33 (15)	5 (33.33)	9 (60.00)	1 (6.67)	9 (60.00)	4 (26.67)	2 (13.33)
B.1.1.28 (15)	9 (60.00)	4 (60.00)	2 (13.33)	6 (40.00)	7 (46.67)	2 (13.33)
B.1.1.411 (4)	2 (50.00)	1 (25.00)	1 (25.00)	1 (25.00)	2 (50.00)	1 (25.00)
B.1.1.161 (4)	1 (25.00)	3 (75.00)	0 (0)	3 (75.00)	1 (25.00)	0 (0)
N.4 (3)	3 (100)	0 (0)	0 (0)	0 (0)	3 (100)	0 (0)
Kappa (1)	1 (100)	0 (0)	0 (0)	0 (0)	1 (100)	0 (0)
B.1.1.54 (1)	0 (0.00)	1 (100)	0 (0)	1 (100)	0 (0)	0 (0)
B.1.1.203 (1)	1 (100)	0 (0)	0 (0)	1 (100)	0 (0)	0 (0)
B.1 (1)	1 (100)	0 (0)	0 (0)	0 (0)	1 (100)	0 (0)

We performed a maximum likelihood phylogenetic analysis using a dataset of 1,034 SARS-CoV-2 nearly complete genome sequences from all five regions of Brazil, including 188 sequences obtained in this study. Our phylogenetic reconstruction indicates that multiple introduction events of different SARS-CoV-2 lineages occurred in the indigenous villages in Mato Grosso do Sul state through time. This data suggests the continuous mobility of people between the villages and urban areas in the Dourados Indigenous Reserve area. The increase in the frequency of SARS-CoV-2 variants detected over time is shown in Figure 3.

### 3.3. SARS-CoV-2 lineages distribution

Analyzing the lineages found in this study and the villages and ethnicities studied here, our data show that the Guarani ethnicity presented a higher incidence of COVID-19 cases (Supplementary Figure 1 and Table 2) (61.70%) and the most prevalent lineage was the Zeta lineage, representing 35.34% of the total cases in this ethnicity, followed by B.1.1 (21.55%), Delta (12.07%) and Gamma (11.21%) lineages. The second ethnicity was the Terena, presenting a total of 63 cases (33.51%). The most prevalent lineage reported in this ethnicity was Delta and B.1.1 lineages, both representing 19.05% of the total cases, followed by Gamma (17.46%) and Zeta (15.87%) lineages. “Other” less representative ethnicities reported a total of 9 cases (4.79%) and mostly reported Gamma (33.33%) and B.1.1.28 (22.22%) lineages.

Regarding the distribution of cases between the indigenous villages described in this study, the Jaguapiru village reported the highest number of COVID-19 cases (54.26%). The most predominant lineages in this village were the Zeta lineage (31.37%), followed by Delta (16.17%), Gamma, and B.1.1 lineages (15·69%). The Bororó village recorded 32.45% of the total cases (61/188) and the most prevalent lineages were B.1.1 (27.87%), Zeta (24.59%), Delta (14.75%) and B.1.1.28 (11.47%). In “other” less representative ethnicities, the Gamma lineage was the most reported (40.0%), followed by Zeta and B.1.1 (20.0%) (Supplementary Figure 2 and Table 2).

## 4. Discussion

Brazilian indigenous peoples have different responses to new diseases, raising the concern about COVID-19 in these populations (25). It is extremely important to obtain information on the incidence of different pathogens that infect indigenous peoples, which is one of the pillars of our study, where we sought to identify and report SARS-CoV-2 variants that circulated within the indigenous villages in Mato Grosso do Sul and correlate it with clinical and sociodemographic data. Our study took place mostly in the Dourados Indigenous Reserve area, which, like other indigenous reserves, is heavily impacted mainly by social inequalities and the spread of communicable diseases (26). We obtained sequences from 188 samples from indigenous peoples, identified 13 different SARS-CoV-2 lineages circulating in the indigenous population from Dourados Indigenous Reserve area, and showed multiple introductions of these lineages in the indigenous reserve area.

During the study period, Brazil reported 22.076.863 cases and 614.186 deaths caused by SARS-CoV-2, and a mortality rate of 2.78%; while MS reported 378.715 confirmed cases and 9.681 deaths, and a mortality rate in the state of 2.56%. According to data from the Ministry of Health, Special Secretary for Indigenous Health (27), from the start of the pandemic in Brazil (March 2020) until February 2023, the number of confirmed cases of COVID-19 among the country’s indigenous peoples was 55,821, and 839 deaths, with a national indigenous mortality rate of 1.50%. The state of Mato Grosso do Sul ranked first in the number of confirmed cases and deaths among indigenous peoples, recording 6.633 cases and 164 deaths (2.47% of mortality rate) (27). In our study, we recorded 1.47% mortality rate (3/204). Although other studies have showed that indigenous people could present a higher mortality rate when compared to non-indigenous population for other infectious diseases (28) we found a slightly lower mortality rate in our population, and it could be due to limitations of this study. The difficulty of accessing indigenous villages and the customs of often not seeking medical care could have contributed to less diagnosis testing in this population, contributing to the underreporting number of COVID-19 cases and deaths, which could also contribute to lower mortality rates (27). The low age structure reported in this population could be a protection for them once it is known that youth tend to have a better response to infection and, consequently, a lower mortality rate when compared to the older adult indigenous population (4). Even though we found a slightly lower mortality rate in our cohort; we should not make this comparison without accounting for the differences cited above.

Despite the genomic surveillance efforts in Brazil, with cases being sequenced as soon as the first confirmed infections were detected in Brazil, there is still a paucity of genomic data from indigenous populations. According to data obtained from GISAID^3^, during period of this study, from January 2020 to March 2023, the number of sequenced and public SARS-CoV-2 sequences was 177,575 out of a total of 36.960.888 confirmed cases of COVID-19 in Brazil. These data show that only 0.515% of positive cases from the general population have been sequenced and shared in Brazil, and none of those are from the indigenous population. In this study, we obtained 188 nearly complete genome sequences, representing 2.83% of COVID-19 cases in this population in Mato Grosso do Sul (204/5,018), a higher percentage if we consider the total of SARS-CoV-2 sequences among the general population in Brazil.

The difficulties that place indigenous peoples in a state of more fragility, such as the lack of basic sanitation, combined with their typical customs, such as housing commonly shared by many indigenous people, the mutual sharing of general-purpose artifacts and personal objects, contribute significantly to viral infection and spread of multiple strains of SARS-CoV-2 in these peoples (29). It is also important to note that indigenous peoples usually face a high incidence of infectious diseases such as malaria and tuberculosis, and vaccine-preventable diseases (28). We noticed during the period of this study a continuous circulation of multiple variants of SARS-CoV-2, possibly because of multiple introduction events and spread of the virus, facilitated by the indigenous’ mobility. Indigenous mobility is more frequent in the Jaguapiru village because it is closer to the city of Dourados, maintaining a continuous circulation among the village and the city, mainly of younger men that are looking for a job or donation in the main city for their subsistence. This fact may have contributed to the fact that most patients that reported having close contact with a suspected or confirmed case were from this village, significantly contributing to the progression and dissemination of the disease in the village.

The pandemic has brought to light several vulnerabilities that indigenous communities face, so when the question of new variants of COVID-19 is brought up we need to increase our attention to this population and how the new scenario will pose a threat to their health and well-being, as they often have limited immunity to diseases introduced from the outside world (28). In this study we identified 13 different lineages circulating among the indigenous population of MS from June 2020 to November 2021. Among those variants, the B.1.1 variant had a greater predominance over the others. Starting in December 2020, the Zeta lineage was the predominant lineage, until March 2021. The Zeta lineage was identified for the first time in October 2020 in Rio de Janeiro state and is described as having the S:E484K mutation, which confers the ability to evade neutralizing antibody responses and was related to reinfection cases (23, 24). Although this variant does not contain mutations that increase its transmissibility potential, it quickly spread across the country (25). In less than 2 months, it was detected in indigenous villages in the countryside of Mato Grosso do Sul state. In our study, the Gamma variant was first detected in March 2021, 2 months after it had been first detected in Brazil, with subsequent predominance over the Zeta variant, remaining predominant until October 2021, when the Delta variant was first detected in this population and took over the predominance of COVID-19 cases in those villages (25). Among the 13 lineages described here, eight (Zeta, Gamma, Delta, B.1.1.411, B.1.1.33, B.1.1.28, B.1.1.161, B.1.1) were present in the Jaguapiru and Bororó villages, located in the Dourados Indigenous Reserve area (Figure 1).

Our results corroborate other studies that describe the same pattern of lineage circulation was also observed in the general population in Brazil (30, 31), also showing a sustained transmission among indigenous peoples. We emphasize that Dourados is the largest city in the countryside of the state and is known as being a connecting city in the route for commerce and agriculture, linking the south of MS state to the rest of Brazil. The virus spread rapidly among indigenous communities, leading to high infection rates and devastating consequences. Other studies have showed disparities and limitations of indigenous population to face COVID-19, when compared to the general population (4, 32–34), corroborating the vulnerability of a population that should be protected.

The COVID-19 pandemic has highlighted the existing inequalities and vulnerabilities faced by indigenous peoples in Brazil. Hence, there is a need to evaluate the strategies to improve the health outcomes of the indigenous population. The comprehensive and culturally sensitive health policies that address the challenges faced by these communities, not only during sanitary crises, are required.

This study presents some limitations, such as the inconsistency or absence of secondary data in public spreadsheets; the scarcity of data on indigenous peoples; the scarcity of sequencing in the literature; the low number of positive samples collected; and the possibility of underreporting of cases and deaths caused by COVID-19 in the targeted population.

The real-time update feature of this platform during the pandemic period posed a limitation as it frequently did not synchronize simultaneously with case notification numbers. Consenquently, numerous patients were left with incomplete data regarding their symptom onset dates, vaccination details, and vaccine coverage. Nevertheless, we addressed this issue by excluding cases with incomplete information from our study.

## 5. Conclusion

In this study, we showed the circulation of multiple SARS-CoV-2 variants in the indigenous population, which should be isolated and protected as they belong to the most fragile group due to their socioeconomic and cultural disparities. Unfortunately, for indigenous peoples, this means an even worse loss since it results in irreparable human and cultural losses. We highlight the inefficiency of protective measures to protect this population from COVID-19 infection and reinforce the importance of paying attention to the less favored and more exposed populations so that public policy decision-makers could quickly and effectively respond in situations that put the Indigenous peoples at risk. We reinforce the need for constant genomic surveillance to monitor and prevent the spread of new emerging viruses and to better understand the viral dynamics in these populations, making it possible to direct specific actions.

## Data availability statement

The datasets presented in this study can be found in online repositories. The names of the repository/repositories and accession number(s) can be found in the article/Supplementary Material.

## Ethics statement

The studies involving human participants were reviewed and approved by Brazilian Research Ethics Committee (CONEP) protocol number 4.502.250. The patients/participants provided their written informed consent to participate in this study. Written informed consent was obtained from the individual(s) for the publication of any potentially identifiable images or data included in this article.

## Author contributions

IR, LO, and VN realized the formal writing of the manuscript and preparation of the original draft. SS, SM, AT, JC, CG, and LA performed to conception and design of the study, supervision, review, and background acquisition. VN and LO contributed with methodology, sociodemographic survey, and organization of the database. DT and MZ performed sample collection. LF, JX, EC, ML, VF, FI, TA, and MG contributed to sequencing performance and statistical analysis. IR performed the genomic analysis. LO and SS received a research grant from CNPq. All authors contributed to the revision of the manuscript, read, and approved the submitted version.

## Footnotes

^1^ https://pangolin.cog-uk.io/

^2^ https://www.gisaid.org/

^3^ www.gisaid.org

## Supplementary material

Supplementary TABLE 1 Information about SARS-CoV-2 sequences generated in this study.

Supplementary Figure 1 A-SARS-CoV-2 lineages according to self-reported ethnicities. B-SARS-CoV-2 lineages according to the gender of patients.

Supplementary Figure 2 Distribution of SARS-CoV-2 lineages related to the total number of positive individuals extracted by indigenous village in the municipality of Dourados - MS.
