# Corrigendum: Genomic characterization of SARS-CoV-2 from an indigenous reserve in Mato Grosso do Sul, Brazil

**DOI:** 10.3389/fpubh.2024.1461598

**Published:** 2024-07-31

**Authors:** Laís Albuquerque de Oliveira, Izabela Mauricio de Rezende, Vinicius João Navarini, Silvana Beutinger Marchioro, Alex José Leite Torres, Julio Croda, Mariana Garcia Croda, Crhistinne Cavalheiro Maymone Gonçalves, Joilson Xavier, Emerson de Castro, Mauricio Lima, Felipe Iani, Talita Adelino, Flávia Aburjaile, Luiz Henrique Ferraz Demarchi, Deborah Ledesma Taira, Marina Castilhos Souza Umaki Zardin, Vagner Fonseca, Marta Giovanetti, Jason Andrews, Luiz Carlos Junior Alcantara, Simone Simionatto

**Affiliations:** ^1^Health Sciences Research Laboratory, Federal University of Grande Dourados, Dourados, Mato Grosso do Sul, Brazil; ^2^Stanford Pandemic Preparedness Hub, Department of Medicine, Division of Infectious Diseases and Geographic Medicine, Stanford University School of Medicine, Stanford, CA, United States; ^3^Laboratory of Immunology and Molecular Biology, Institute of Health Sciences, Federal University of Bahia, Salvador, Bahia, Brazil; ^4^Oswaldo Cruz Foundation, Campo Grande, Mato Grosso do Sul, Brazil; ^5^Faculdade de Medicina (FAMED), Universidade Federal do Mato Grosso do Sul, Campo Grande, Mato Grosso do Sul, Brazil; ^6^School of Medicine, Federal University of Mato Grosso do Sul, Campo Grande, Mato Grosso do Sul, Brazil; ^7^State Secretariat of Health of Mato Grosso do Sul, Campo Grande, Mato Grosso do Sul, Brazil; ^8^Federal University of Minas Gerais, Belo Horizonte, Minas Gerais, Brazil; ^9^Ezequiel Dias Foundation (FUNED), Belo Horizonte, Minas Gerais, Brazil; ^10^Preventive Veterinary Medicine Departament, Veterinary School, Universidade Federal de Minas Gerais, Belo Horizonte, Brazil; ^11^Central Public Health Laboratory (Lacen), Campo Grande, Mato Grosso do Sul, Brazil; ^12^Pan American Health Organization - PAHO, Brasília, Distrito Federal, Brazil; ^13^Rene Rachou, Fundação Oswaldo Cruz, Belo Horizonte, Minas Gerais, Brazil; ^14^Sciences and Technologies for Sustainable Development and One Health, Università Campus Bio-Medico di Roma, Rome, Italy; ^15^Climate-Amplified Diseases and Epidemics (CLIMADE) Rio de Janeiro, Rio de Janeiro, Brazil

**Keywords:** SARS-CoV-2, COVID-19, indigenous population, VoI, VOC, pandemic

In the published article, there was an error in Figure 3 as published. The incorrect version of the figure was published. The corrected Figure 3 and its caption appear below.

**Figure 3:**
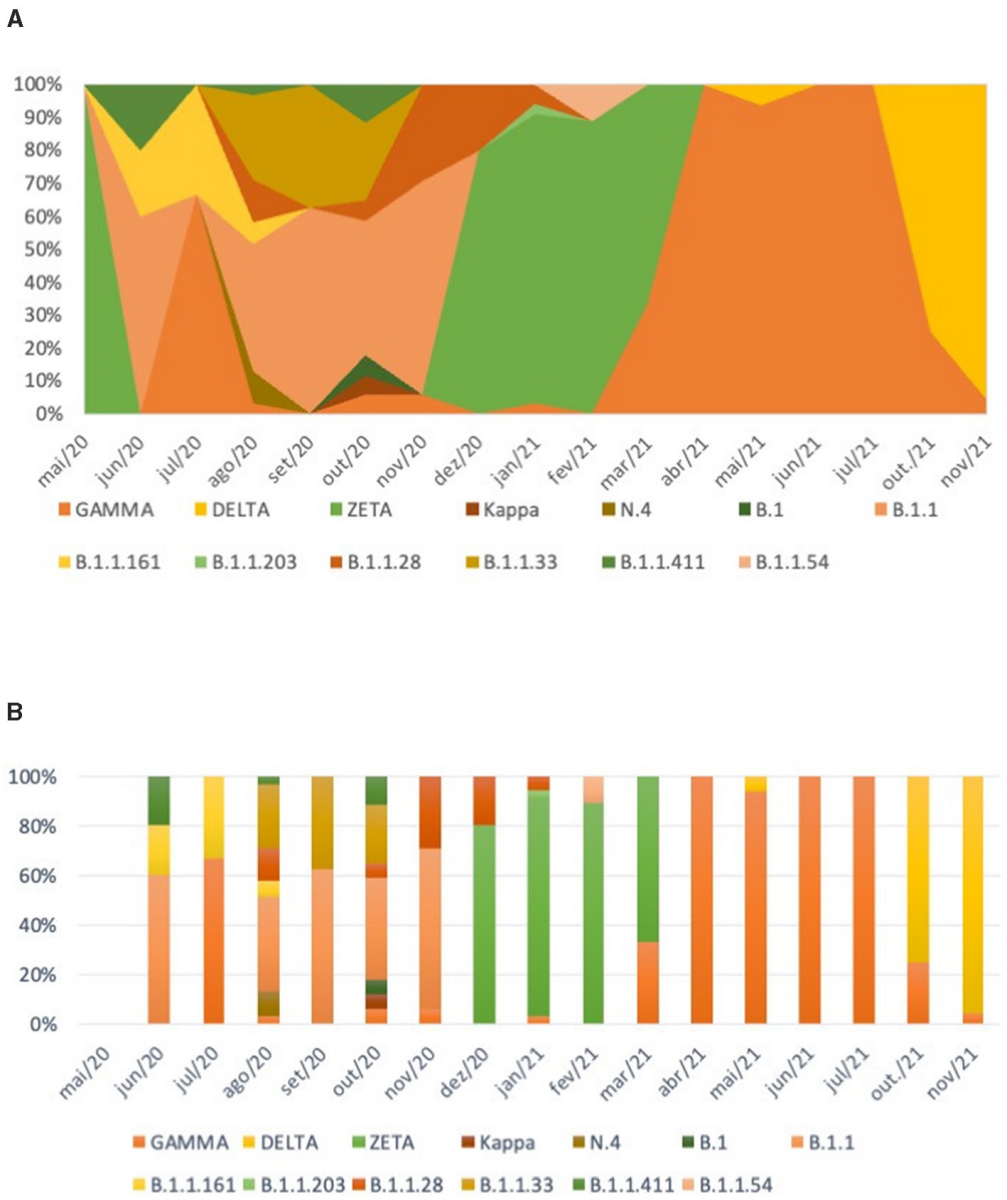
Distribution of SARS-CoV-2 variants over time. **(A)** Distribution of SARS-CoV-2 Pangolin strains according to symptom onset. **(B)** Distribution of SARS-CoV-2 strains according to the nomenclature proposed by the WHO Technical Advisory.

The authors apologize for this error and state that this does not change the scientific conclusions of the article in any way. The original article has been updated.

