# Supplementary material for: Genomic characterization of SARS-CoV-2 from an indigenous reserve in Mato Grosso do Sul, Brazil
Source: Front Public Health. 2023 Oct 26;11:1195779. doi: 10.3389/fpubh.2023.1195779 (PMC10641392; doi:10.3389/fpubh.2023.1195779)
Supplement: Supplementary TABLE 1 — Information about SARS-CoV-2 sequences generated in this study. [file Table_1.pdf]

Supplementary table 1 – Information about SARS-CoV-2 sequences generated in this study.

ID FUNED	GISAID ID	Collecting date (yyyy-MM-dd)	SARS-CoV-2 variants (Pangolin classification)	Municipality	Indigenous village	Indigenous ethnicity
1	hCoV-19_Brazil_MS-GD01_2021	2021-03-08	Zeta (P.2)	DOURADOS	BORORÓ	TERENA
2	hCoV-19_Brazil_MS-GD02_2021	2021-03-09	Zeta (P.2)	DOURADOS	BORORÓ	GUARANI KAIOWÁ
3	hCoV-19_Brazil_MS-GD03_2021	2021-02-04	Zeta (P.2)	DOURADOS	JAGUAPIRÚ	GUARANI KAIOWÁ
4	hCoV-19_Brazil_MS-GD04_2021	2021-01-29	Zeta (P.2)	DOURADOS	JAGUAPIRÚ	TERENA
6	hCoV-19_Brazil_MS-GD06_2021	2021-02-02	Zeta (P.2)	DOURADOS	BORORÓ	GUARANI
7	hCoV-19_Brazil_MS-GD07_2021	2021-02-08	Zeta (P.2)	DOURADOS	PANAMBIZINHO	GUARANI KAIOWÁ
8	hCoV-19_Brazil_MS-GD08_2021	2021-02-09	Zeta (P.2)	DOURADOS	PANAMBIZINHO	GUARANI KAIOWÁ
9	hCoV-19_Brazil_MS-GD09_2021	2021-02-10	Zeta (P.2)	DOURADOS	BORORÓ	GUARANI KAIOWÁ
10	hCoV-19_Brazil_MS-GD10_2021	2021-02-09	Zeta (P.2)	DOURADOS	BORORÓ	GUARANI KAIOWÁ
11	hCoV-19_Brazil_MS-GD11_2021	2021-02-11	Zeta (P.2)	MIRANDA	1MOREIRA	TERENA
12	hCoV-19_Brazil_MS-GD12_2021	2021-02-18	Other	DOURADOS	JAGUAPIRÚ	TERENA
13	hCoV-19_Brazil_MS-GD13_2021	2021-02-18	Zeta (P.2)	DOURADOS	JAGUAPIRÚ	TERENA
14	hCoV-19_Brazil_MS-GD14_2021	2021-01-25	Zeta (P.2)	DOURADOS	JAGUAPIRÚ	GUARANI KAIOWÁ
15	hCoV-19_Brazil_MS-GD15_2021	2021-01-22	Zeta (P.2)	DOURADOS	BORORÓ	GUARANI KAIOWÁ
16	hCoV-19_Brazil_MS-GD16_2021	2021-01-21	Zeta (P.2)	DOURADOS	JAGUAPIRÚ	GUARANI KAIOWÁ
17	hCoV-19_Brazil_MS-GD17_2021	2021-01-21	Zeta (P.2)	DOURADOS	JAGUAPIRÚ	GUARANI KAIOWÁ
18	hCoV-19_Brazil_MS-GD18_2021	2021-01-21	Zeta (P.2)	DOURADOS	JAGUAPIRÚ	GUARANI KAIOWÁ
19	hCoV-19_Brazil_MS-GD19_2021	2021-01-21	Zeta (P.2)	DOURADOS	JAGUAPIRÚ	GUARANI KAIOWÁ
20	hCoV-19_Brazil_MS-GD20_2021	2021-01-20	Zeta (P.2)	TACURU	1SASSORÓ	GUARANI KAIOWÁ
21	hCoV-19_Brazil_MS-GD21_2021	2021-01-20	Gamma (P.1)	DOURADOS	JAGUAPIRÚ	TERENA
22	hCoV-19_Brazil_MS-GD22_2021	2021-01-19	Other	DOURADOS	JAGUAPIRÚ	GUARANI KAIOWÁ
24	hCoV-19_Brazil_MS-GD24_2021	2021-01-18	Zeta (P.2)	DOURADOS	JAGUAPIRÚ	GUARANI KAIOWÁ

25	hCoV-19_Brazil_MS-GD25_2021	2021-01-12	Zeta (P.2)	DOURADOS	JAGUAPIRÚ	GUARANI KAIOWÁ
26	hCoV-19_Brazil_MS-GD26_2021	2021-01-12	Zeta (P.2)	DOURADOS	JAGUAPIRÚ	GUARANI
27	hCoV-19_Brazil_MS-GD27_2020	2020-10-13	N.9	DOURADOS	BORORÓ	GUARANI NHANDEVA
28	hCoV-19_Brazil_MS-GD28_2021	2021-01-14	Zeta (P.2)	DOURADOS	JAGUAPIRÚ	GUARANI KAIOWÁ
29	hCoV-19_Brazil_MS-GD29_2021	2021-01-15	Zeta (P.2)	DOURADOS	JAGUAPIRÚ	TERENA
30	hCoV-19_Brazil_MS-GD30_2021	2021-01-14	Zeta (P.2)	DOURADOS	JAGUAPIRÚ	TERENA
31	hCoV-19_Brazil_MS-GD31_2020	2020-10-20	Other	DOURADOS	BORORÓ	GUARANI KAIOWÁ
32	hCoV-19_Brazil_MS-GD32_2020	2020-10-06	N.9	DOURADOS	JAGUAPIRÚ	GUARANI KAIOWÁ
33	hCoV-19_Brazil_MS-GD33_2020	2020-10-14	N.9	DOURADOS	BORORÓ	GUARANI KAIOWÁ
34	hCoV-19_Brazil_MS-GD34_2020	2020-09-11	N.9	DOURADOS	JAGUAPIRÚ	TERENA
35	hCoV-19_Brazil_MS-GD35_2020	2020-09-09	N.9	DOURADOS	ALTOS INDAIA	N/A
36	hCoV-19_Brazil_MS-GD36_2020	2020-09-04	N.9	DOURADOS	JAGUAPIRÚ	TERENA
37	hCoV-19_Brazil_MS-GD37_2020	2020-09-04	N.9	DOURADOS	JAGUAPIRÚ	TERENA
38	hCoV-19_Brazil_MS-GD38_2020	2020-09-04	N.9	DOURADOS	JAGUAPIRÚ	TERENA
39	hCoV-19_Brazil_MS-GD39_2020	2020-09-04	B.1.1.28	DOURADOS	JAGUAPIRÚ	GUARANI KAIOWÁ
40	hCoV-19_Brazil_MS-GD40_2020	2020-09-03	B.1.1.28	DOURADOS	JAGUAPIRÚ	TERENA
41	hCoV-19_Brazil_MS-GD41_2020	2020-08-31	N.9	DOURADOS	BORORÓ	GUARANI KAIOWÁ
42	hCoV-19_Brazil_MS-GD42_2020	2020-08-31	N.9	DOURADOS	BORORÓ	GUARANI KAIOWÁ
43	hCoV-19_Brazil_MS-GD43_2020	2020-09-01	N.9	DOURADOS	BORORÓ	GUARANI KAIOWÁ
44	hCoV-19_Brazil_MS-GD44_2020	2020-08-31	N.9	DOURADOS	JAGUAPIRÚ	TERENA
45	hCoV-19_Brazil_MS-GD45_2020	2020-08-31	B.1.1.28	DOURADOS	JAGUAPIRÚ	GUARANI KAIOWÁ
46	hCoV-19_Brazil_MS-GD46_2020	2020-08-31	N.9	DOURADOS	JAGUAPIRÚ	TERENA
47	hCoV-19_Brazil_MS-GD47_2020	2020-08-28	N.9	DOURADOS	BORORÓ	GUARANI KAIOWÁ
48	hCoV-19_Brazil_MS-GD48_2020	2020-08-28	N.9	DOURADOS	BORORÓ	GUARANI
49	hCoV-19_Brazil_MS-GD49_2020	2020-09-11	B.1.1	DOURADOS	JAGUAPIRÚ	TERENA
50	hCoV-19_Brazil_MS-GD50_2020	2020-09-11	N.9	DOURADOS	JAGUAPIRÚ	TERENA

51	hCoV-19_Brazil_MS-GD51_2020	2020-08-26	B.1.1.28	DOURADOS	JAGUAPIRÚ	TERENA
52	hCoV-19_Brazil_MS-GD52_2020	2020-08-21	B.1.1	MIRANDA	1LALIMA	TERENA
53	hCoV-19_Brazil_MS-GD53_2020	2020-08-21	N.9	DOURADOS	JAGUAPIRÚ	TERENA
54	hCoV-19_Brazil_MS-GD54_2020	2020-08-21	B.1.1	DOURADOS	JAGUAPIRÚ	TERENA
55	hCoV-19_Brazil_MS-GD55_2020	2020-08-19	Other	DOURADOS	JAGUAPIRÚ	TERENA
56	hCoV-19_Brazil_MS-GD56_2020	2020-08-17	B.1.1	DOURADOS	JAGUAPIRÚ	TERENA
57	hCoV-19_Brazil_MS-GD57_2020	2020-08-17	B.1.1	DOURADOS	JAGUAPIRÚ	GUARANI KAIOWÁ
58	hCoV-19_Brazil_MS-GD58_2020	2020-08-17	B.1.1	DOURADOS	JAGUAPIRÚ	TERENA
59	hCoV-19_Brazil_MS-GD59_2020	2020-08-14	B.1.1	DOURADOS	ACAMPAMENTOS	GUARANI
60	hCoV-19_Brazil_MS-GD60_2020	2020-08-14	Other	DOURADOS	JAGUAPIRÚ	TERENA
61	hCoV-19_Brazil_MS-GD61_2020	2020-08-14	B.1.1	DOURADOS	JAGUAPIRÚ	TERENA
62	hCoV-19_Brazil_MS-GD62_2020	2020-08-14	B.1.1	DOURADOS	JAGUAPIRÚ	TERENA
63	hCoV-19_Brazil_MS-GD63_2020	2020-08-11	B.1.1	DOURADOS	JAGUAPIRÚ	TERENA
64	hCoV-19_Brazil_MS-GD64_2020	2020-08-11	B.1.1	DOURADOS	JAGUAPIRÚ	TERENA
65	hCoV-19_Brazil_MS-GD65_2020	2020-08-06	B.1.1	DOURADOS	JAGUAPIRÚ	GUARANI
66	hCoV-19_Brazil_MS-GD66_2020	2020-08-11	B.1.1	DOURADOS	JAGUAPIRÚ	TERENA
67	hCoV-19_Brazil_MS-GD67_2020	2020-08-06	Other	DOURADOS	JAGUAPIRÚ	TERENA
69	hCoV-19_Brazil_MS-GD69_2020	2020-07-21	Other	DOURADOS	JAGUAPIRÚ	TERENA
70	hCoV-19_Brazil_MS-GD70_2020	2020-07-02	B.1.1	DOURADOS	BORORÓ	GUARANI KAIOWÁ
71	hCoV-19_Brazil_MS-GD71_2020	2020-06-23	B.1.1	DOURADOS	JAGUAPIRÚ	GUARANI KAIOWÁ
73	hCoV-19_Brazil_MS-GD73_2020	2020-07-02	B.1.1	DOURADOS	BORORÓ	GUARANI KAIOWÁ
74	hCoV-19_Brazil_MS-GD74_2020	2020-10-20	B.1.1	DOURADOS	BORORÓ	GUARANI KAIOWÁ
75	hCoV-19_Brazil_MS-GD75_2020	2020-10-23	B.1.1	DOURADOS	JAGUAPIRÚ	GUARANI KAIOWÁ
76	hCoV-19_Brazil_MS-GD76_2020	2020-10-23	Other	DOURADOS	BORORÓ	GUARANI KAIOWÁ
77	hCoV-19_Brazil_MS-GD77_2020	2020-10-23	B.1.1	DOURADOS	BORORÓ	GUARANI KAIOWÁ
78	hCoV-19_Brazil_MS-GD78_2020	2020-10-23	N.9	DOIS IRMAOS DO BURITI	OLHO DAGUA	TERENA

79	hCoV-19_Brazil_MS-GD79_2020	2020-10-28	B.1.1	DOURADOS	BORORÓ	GUARANI KAIOWÁ
80	hCoV-19_Brazil_MS-GD80_2020	2020-10-23	B.1.1	DOURADOS	BORORÓ	GUARANI KAIOWÁ
81	hCoV-19_Brazil_MS-GD81_2020	2020-10-29	B.1.1	DOURADOS	BORORÓ	GUARANI KAIOWÁ
82	hCoV-19_Brazil_MS-GD82_2020	2020-11-03	B.1.1.28	DOURADOS	BORORÓ	GUARANI KAIOWÁ
83	hCoV-19_Brazil_MS-GD83_2020	2020-11-05	B.1.1.28	DOURADOS	BORORÓ	GUARANI KAIOWÁ
84	hCoV-19_Brazil_MS-GD84_2020	2020-11-06	B.1.1.28	DOURADOS	BORORÓ	GUARANI KAIOWÁ
85	hCoV-19_Brazil_MS-GD85_2020	2020-11-06	B.1.1.28	DOURADOS	BORORÓ	TERENA
86	hCoV-19_Brazil_MS-GD86_2020	2020-11-11	B.1.1	CAARAPÓ	CAARAPÓ	GUARANI KAIOWÁ
87	hCoV-19_Brazil_MS-GD87_2020	2020-11-11	B.1.1	DOURADOS	BORORÓ	GUARANI KAIOWÁ
88	hCoV-19_Brazil_MS-GD88_2020	2020-11-11	B.1.1.28	DOURADOS	BORORÓ	GUARANI
89	hCoV-19_Brazil_MS-GD89_2020	2020-11-11	B.1.1.28	AQUIDAUANA	LIMÃO VERDE	TERENA
90	hCoV-19_Brazil_MS-GD90_2021	2021-01-14	B.1.1.28	DOURADOS	JAGUAPIRÚ	GUARANI KAIOWÁ
91	hCoV-19_Brazil_MS-GD91_2020	2020-11-16	B.1.1	DOURADOS	BORORÓ	GUARANI KAIOWÁ
92	hCoV-19_Brazil_MS-GD92_2020	2020-11-20	B.1.1	DOURADOS	BORORÓ	GUARANI KAIOWÁ
93	hCoV-19_Brazil_MS-GD93_2020	2020-11-20	B.1.1	DOURADOS	BORORÓ	GUARANI KAIOWÁ
94	hCoV-19_Brazil_MS-GD94_2020	2020-11-20	B.1.1	DOURADOS	BORORÓ	GUARANI KAIOWÁ
95	hCoV-19_Brazil_MS-GD95_2020	2020-11-20	B.1.1	DOURADOS	BORORÓ	GUARANI KAIOWÁ
96	hCoV-19_Brazil_MS-GD96_2020	2020-11-23	B.1.1	DOURADOS	BORORÓ	GUARANI KAIOWÁ
97	hCoV-19_Brazil_MS-GD97_2020	2020-11-23	B.1.1	DOURADOS	BORORÓ	GUARANI
98	hCoV-19_Brazil_MS-GD98_2020	2020-11-24	B.1.1	DOURADOS	BORORÓ	GUARANI KAIOWÁ
99	hCoV-19_Brazil_MS-GD99_2020	2020-12-10	Zeta (P.2)	DOURADOS	BORORÓ	GUARANI KAIOWÁ
100	hCoV-19_Brazil_MS-GD100_2020	2020-12-10	Zeta (P.2)	DOURADOS	JAGUAPIRÚ	TERENA
101	hCoV-19_Brazil_MS-GD101_2020	2020-12-14	B.1.1.28	ITAPORÃ	CENTRO	N/A
102	hCoV-19_Brazil_MS-GD102_2020	2020-12-16	Zeta (P.2)	DOURADOS	BORORÓ	GUARANI KAIOWÁ
103	hCoV-19_Brazil_MS-GD103_2020	2020-12-16	Zeta (P.2)	DOURADOS	BORORÓ	GUARANI NHANDEVA
104	hCoV-19_Brazil_MS-GD104_2020	2020-12-17	B.1.1.28	DOURADOS	BORORÓ	GUARANI KAIOWÁ

105	hCoV-19_Brazil_MS-GD105_2020	2020-11-23	B.1.1	DOURADOS	BORORÓ	GUARANI KAIOWÁ
106	hCoV-19_Brazil_MS-GD106_2021	2021-01-06	B.1.1.28	DOURADOS	JAGUAPIRÚ	N/a
107	hCoV-19_Brazil_MS-GD107_2021	2021-01-06	Zeta (P.2)	DOURADOS	BORORÓ	GUARANI KAIOWÁ
108	hCoV-19_Brazil_MS-GD108_2021	2021-01-06	Zeta (P.2)	DOURADOS	JAGUAPIRÚ	GUARANI
109	hCoV-19_Brazil_MS-GD109_2021	2021-01-06	B.1.1.28	DOURADOS	BORORÓ	GUARANI
110	hCoV-19_Brazil_MS-GD110_2021	2021-01-07	Zeta (P.2)	DOURADOS	JAGUAPIRÚ	GUARANI KAIOWÁ
111	hCoV-19_Brazil_MS-GD111_2021	2021-01-07	Zeta (P.2)	DOURADOS	JAGUAPIRÚ	GUARANI KAIOWÁ
112	hCoV-19_Brazil_MS-GD112_2020	2020-10-08	B.1.1	DOURADOS	JAGUAPIRÚ	GUARANI KAIOWÁ
113	hCoV-19_Brazil_MS-GD113_2020	2020-09-11	B.1.1	DOURADOS	JAGUAPIRÚ	TERENA
114	hCoV-19_Brazil_MS-GD114_2020	2020-09-11	B.1.1	DOURADOS	JAGUAPIRÚ	TERENA
115	hCoV-19_Brazil_MS-GD115_2020	2020-09-11	B.1.1	CAARAPÓ	CAARAPÓ	GUARANI KAIOWÁ
116	hCoV-19_Brazil_MS-GD116_2020	2020-10-01	B.1.1	DOURADOS	N/A	N/A
117	hCoV-19_Brazil_MS-GD117_2020	2020-10-23	B.1	DOURADOS	BORORÓ	GUARANI KAIOWÁ
118	hCoV-19_Brazil_MS-GD118_2020	2020-06-25	Other	DOURADOS	1LAGOA RICA	N/A
119	hCoV-19_Brazil_MS-GD119_2021	2021-01-11	Zeta (P.2)	DOURADOS	JAGUAPIRÚ	GUARANI KAIOWÁ
120	hCoV-19_Brazil_MS-GD120_2021	2021-01-11	Zeta (P.2)	DOURADOS	JAGUAPIRÚ	GUARANI KAIOWÁ
121	hCoV-19_Brazil_MS-GD121_2021	2021-01-11	Zeta (P.2)	DOURADOS	JAGUAPIRÚ	GUARANI KAIOWÁ
122	hCoV-19_Brazil_MS-GD122_2021	2021-01-11	Zeta (P.2)	DOURADOS	JAGUAPIRÚ	GUARANI KAIOWÁ
123	hCoV-19_Brazil_MS-GD123_2020	2020-06-15	Other	DOURADOS	BORORÓ	GUARANI KAIOWÁ
125	hCoV-19_Brazil_MS-GD125_2020	2020-10-23	Other	DOURADOS	BORORÓ	GUARANI KAIOWÁ
126	hCoV-19_Brazil_MS-GD126_2021	2021-01-12	Zeta (P.2)	DOURADOS	BORORÓ	GUARANI KAIOWÁ
127	hCoV-19_Brazil_MS-GD127_2021	2021-01-12	Zeta (P.2)	DOURADOS	JAGUAPIRÚ	GUARANI KAIOWÁ
130	hCoV-19_Brazil_MS-GD130_2021	2021-01-11	Zeta (P.2)	DOURADOS	JAGUAPIRÚ	TERENA
131	hCoV-19_Brazil_MS-GD131_2021	2021-01-11	Zeta (P.2)	DOURADOS	JAGUAPIRÚ	GUARANI KAIOWÁ
133	hCoV-19_Brazil_MS-GD133_2021	2021-01-04	Zeta (P.2)	DOURADOS	BORORÓ	TERENA
134	hCoV-19_Brazil_MS-GD134_2020	2020-12-30	Zeta (P.2)	DOURADOS	BORORÓ	TERENA

135	hCoV-19_Brazil_MS-GD135_2020	2020-12-30	Zeta (P.2)	DOURADOS	JAGUAPIRÚ	GUARANI KAIOWÁ
136	hCoV-19_Brazil_MS-GD136_2020	2020-12-30	Zeta (P.2)	DOURADOS	JAGUAPIRÚ	GUARANI KAIOWÁ
137	hCoV-19_Brazil_MS-GD137_2021	2021-01-02	Zeta (P.2)	DOURADOS	JAGUAPIRÚ	GUARANI KAIOWÁ
138	hCoV-19_Brazil_MS-GD138_2020	2020-12-30	Zeta (P.2)	DOURADOS	BORORÓ	GUARANI KAIOWÁ
139	hCoV-19_Brazil_MS-GD139_2020	2020-12-30	Zeta (P.2)	DOURADOS	JAGUAPIRÚ	GUARANI KAIOWÁ
140	hCoV-19_Brazil_MS-GD140_2021	2021-01-18	Zeta (P.2)	DOURADOS	JAGUAPIRÚ	GUARANI KAIOWÁ
141	hCoV-19_Brazil_MS-GD141_2021	2021-02-04	Zeta (P.2)	DOURADOS	JAGUAPIRÚ	GUARANI KAIOWÁ
142	hCoV-19_Brazil_MS-GD142_2021	2021-02-08	Zeta (P.2)	DOURADOS	JARDIM PIRATININGA	OTHERS
144	hCoV-19_Brazil_MS-GD144_2021	2021-01-27	Zeta (P.2)	DOURADOS	JAGUAPIRÚ	GUARANI KAIOWÁ
145	hCoV-19_Brazil_MS-GD145_2021	2021-02-12	Zeta (P.2)	DOURADOS	BORORÓ	GUARANI KAIOWÁ
146	hCoV-19_Brazil_MS-GD146_2021	2021-03-18	Gamma (P.1)	DOURADOS	JAGUAPIRÚ	GUARANI KAIOWÁ
147	hCoV-19_Brazil_MS-GD147_2021	2021-03-19	Gamma (P.1)	DOURADOS	BOQUERÓN	GUARANI KAIOWÁ
149	hCoV-19_Brazil_MS-GD149_2021	2021-04-08	Gamma (P.1)	DOURADOS	JAGUAPIRÚ	GUARANI KAIOWÁ
150	hCoV-19_Brazil_MS-GD150_2021	2021-05-27	Gamma (P.1)	DOURADOS	JARDIM ÁGUA BOA	GUARANI KAIOWÁ
152	hCoV-19_Brazil_MS-GD152_2021	2021-05-10	Gamma (P.1)	DOURADOS	JOQUEI CLUBE	OTHERS
153	hCoV-19_Brazil_MS-GD153_2021	2021-05-14	Gamma (P.1)	CAARAPÓ	CAARAPÓ	GUARANI KAIOWÁ
154	hCoV-19_Brazil_MS-GD154_2021	2021-05-14	Gamma (P.1)	DOURADOS	JOQUEI CLUBE	OTHERS
155	hCoV-19_Brazil_MS-GD155_2021	2021-05-17	Gamma (P.1)	DOURADOS	JAGUAPIRÚ	GUARANI
156	hCoV-19_Brazil_MS-GD156_2021	2021-05-17	Gamma (P.1)	DOURADOS	JAGUAPIRÚ	TERENA
157	hCoV-19_Brazil_MS-GD157_2021	2021-05-17	Gamma (P.1)	DOURADOS	JAGUAPIRÚ	GUARANI
158	hCoV-19_Brazil_MS-GD158_2021	2021-05-21	Gamma (P.1)	DOURADOS	JAGUAPIRÚ	TERENA
160	hCoV-19_Brazil_MS-GD160_2021	2021-05-20	Gamma (P.1)	DOURADOS	VILA INDIO	OTHERS
161	hCoV-19_Brazil_MS-GD161_2021	2021-05-20	Gamma (P.1)	MIRANDA	ARGOLA	TERENA
162	hCoV-19_Brazil_MS-GD162_2021	2021-05-25	Gamma (P.1)	DOURADOS	JAGUAPIRÚ	TERENA
163	hCoV-19_Brazil_MS-GD163_2021	2021-05-26	Gamma (P.1)	DOURADOS	JAGUAPIRÚ	TERENA
164	hCoV-19_Brazil_MS-GD164_2021	2021-05-26	Gamma (P.1)	DOURADOS	JAGUAPIRÚ	TERENA

165	hCoV-19_Brazil_MS-GD165_2021	2021-05-26	Gamma (P.1)	DOURADOS	JAGUAPIRÚ	GUARANI
167	hCoV-19_Brazil_MS-GD167_2021	2021-05-28	Gamma (P.1)	DOURADOS	JAGUAPIRÚ	TERENA
168	hCoV-19_Brazil_MS-GD168_2021	2021-05-28	Gamma (P.1)	DOURADOS	JAGUAPIRÚ	TERENA
169	hCoV-19_Brazil_MS-GD169_2021	2021-05-28	Gamma (P.1)	DOURADOS	JAGUAPIRÚ	TERENA
171	hCoV-19_Brazil_MS-GD171_2021	2021-06-01	Gamma (P.1)	DOURADOS	JAGUAPIRÚ	TERENA
173	hCoV-19_Brazil_MS-GD173_2021	2021-06-01	Gamma (P.1)	DOURADOS	PANAMBIZINHO	GUARANI KAIOWÁ
174	hCoV-19_Brazil_MS-GD174_2021	2021-06-06	Gamma (P.1)	DOURADOS	BORORÓ	GUARANI KAIOWÁ
175	hCoV-19_Brazil_MS-GD175_2021	2021-06-09	Gamma (P.1)	DOURADOS	JAGUAPIRÚ	GUARANI KAIOWÁ
177	hCoV-19_Brazil_MS-GD177_2021	2021-05-27	Gamma (P.1)	DOURADOS	JAGUAPIRÚ	TERENA
178	hCoV-19_Brazil_MS-GD178_2021	2021-07-14	Gamma (P.1)	DOURADOS	JAGUAPIRÚ	GUARANI KAIOWÁ
179	hCoV-19_Brazil_MS-GD179_2021	2021-10-06	Other	DOURADOS	JAGUAPIRÚ	GUARANI KAIOWÁ
180	hCoV-19_Brazil_MS-GD180_2021	2021-10-29	Other	DOURADOS	JAGUAPIRÚ	GUARANI
181	hCoV-19_Brazil_MS-GD181_2021	2021-11-01	Other	DOURADOS	JAGUAPIRÚ	TERENA
182	hCoV-19_Brazil_MS-GD182_2021	2021-11-01	Other	DOURADOS	JAGUAPIRÚ	GUARANI KAIOWÁ
183	hCoV-19_Brazil_MS-GD183_2021	2021-11-01	Other	DOURADOS	BORORÓ	GUARANI KAIOWÁ
184	hCoV-19_Brazil_MS-GD184_2021	2021-05-31	Other	DOURADOS	BORORÓ	GUARANI KAIOWÁ
185	hCoV-19_Brazil_MS-GD185_2021	2021-11-09	Other	DOURADOS	JAGUAPIRÚ	TERENA
186	hCoV-19_Brazil_MS-GD186_2021	2021-11-09	Other	DOURADOS	JAGUAPIRÚ	TERENA
187	hCoV-19_Brazil_MS-GD187_2021	2021-11-10	Other	DOURADOS	JAGUAPIRÚ	TERENA
188	hCoV-19_Brazil_MS-GD188_2021	2021-11-04	Other	DOURADOS	JAGUAPIRÚ	GUARANI
189	hCoV-19_Brazil_MS-GD189_2021	2021-11-04	Other	DOURADOS	JAGUAPIRÚ	GUARANI
190	hCoV-19_Brazil_MS-GD190_2021	2021-11-10	Other	DOURADOS	BORORÓ	GUARANI KAIOWÁ
191	hCoV-19_Brazil_MS-GD191_2021	2021-11-10	Other	DOURADOS	JAGUAPIRÚ	GUARANI KAIOWÁ
192	hCoV-19_Brazil_MS-GD192_2021	2021-11-16	Other	DOURADOS	JAGUAPIRÚ	TERENA
193	hCoV-19_Brazil_MS-GD193_2021	2021-11-17	Other	DOURADOS	JAGUAPIRÚ	GUARANI KAIOWÁ
194	hCoV-19_Brazil_MS-GD194_2021	2021-11-17	Other	DOURADOS	BORORÓ	GUARANI KAIOWÁ

195	hCoV-19_Brazil_MS-GD195_2021	2021-11-17	Other	DOURADOS	BORORÓ	TERENA
196	hCoV-19_Brazil_MS-GD196_2021	2021-11-18	Other	DOURADOS	BORORÓ	TERENA
197	hCoV-19_Brazil_MS-GD197_2021	2021-11-18	Other	DOURADOS	BORORÓ	TERENA
198	hCoV-19_Brazil_MS-GD198_2021	2021-11-18	Other	DOURADOS	BORORÓ	TERENA
199	hCoV-19_Brazil_MS-GD199_2021	2021-11-18	Other	DOURADOS	JAGUAPIRÚ	GUARANI KAIOWÁ
200	hCoV-19_Brazil_MS-GD200_2021	2021-11-18	Other	DOURADOS	JAGUAPIRÚ	TERENA
201	hCoV-19_Brazil_MS-GD201_2021	2021-11-19	Other	DOURADOS	JAGUAPIRÚ	TERENA
202	hCoV-19_Brazil_MS-GD202_2021	2021-11-22	Other	DOURADOS	BORORÓ	GUARANI KAIOWÁ
203	hCoV-19_Brazil_MS-GD203_2021	2021-11-22	Other	DOURADOS	BORORÓ	GUARANI KAIOWÁ
204	hCoV-19_Brazil_MS-GD204_2021	2021-11-23	Other	DOURADOS	BORORÓ	GUARANI KAIOWÁ
