# Supplementary figures and images for: Genomic characterization of SARS-CoV-2 from an indigenous reserve in Mato Grosso do Sul, Brazil
Source: Front Public Health. 2023 Oct 26;11:1195779. doi: 10.3389/fpubh.2023.1195779 (PMC10641392; doi:10.3389/fpubh.2023.1195779)

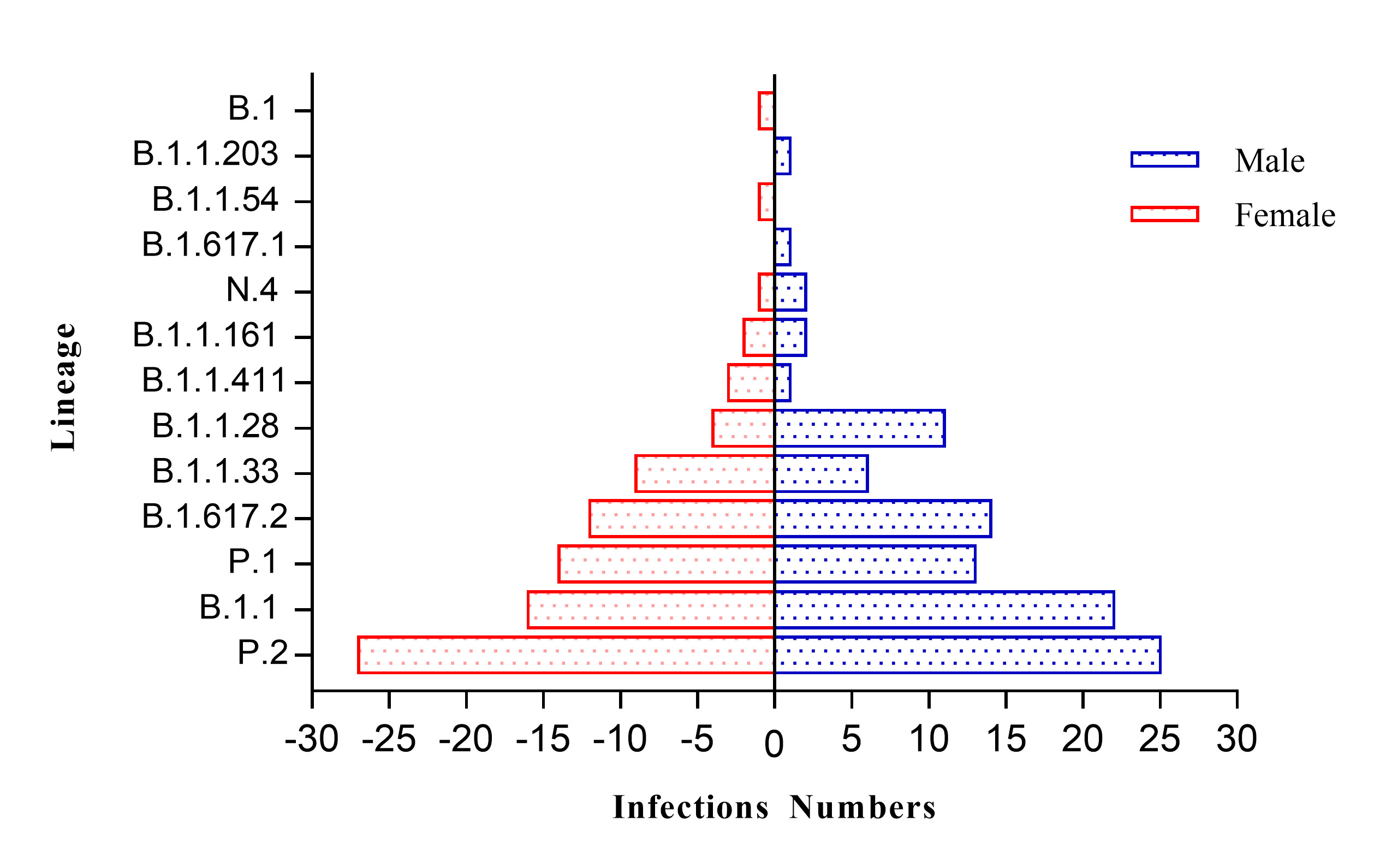

Supplement: Supplementary Figure 1 — A-SARS-CoV-2 lineages according to self-reported ethnicities. B-SARS-CoV-2 lineages according to the gender of patients. [file Image_1.jpeg]

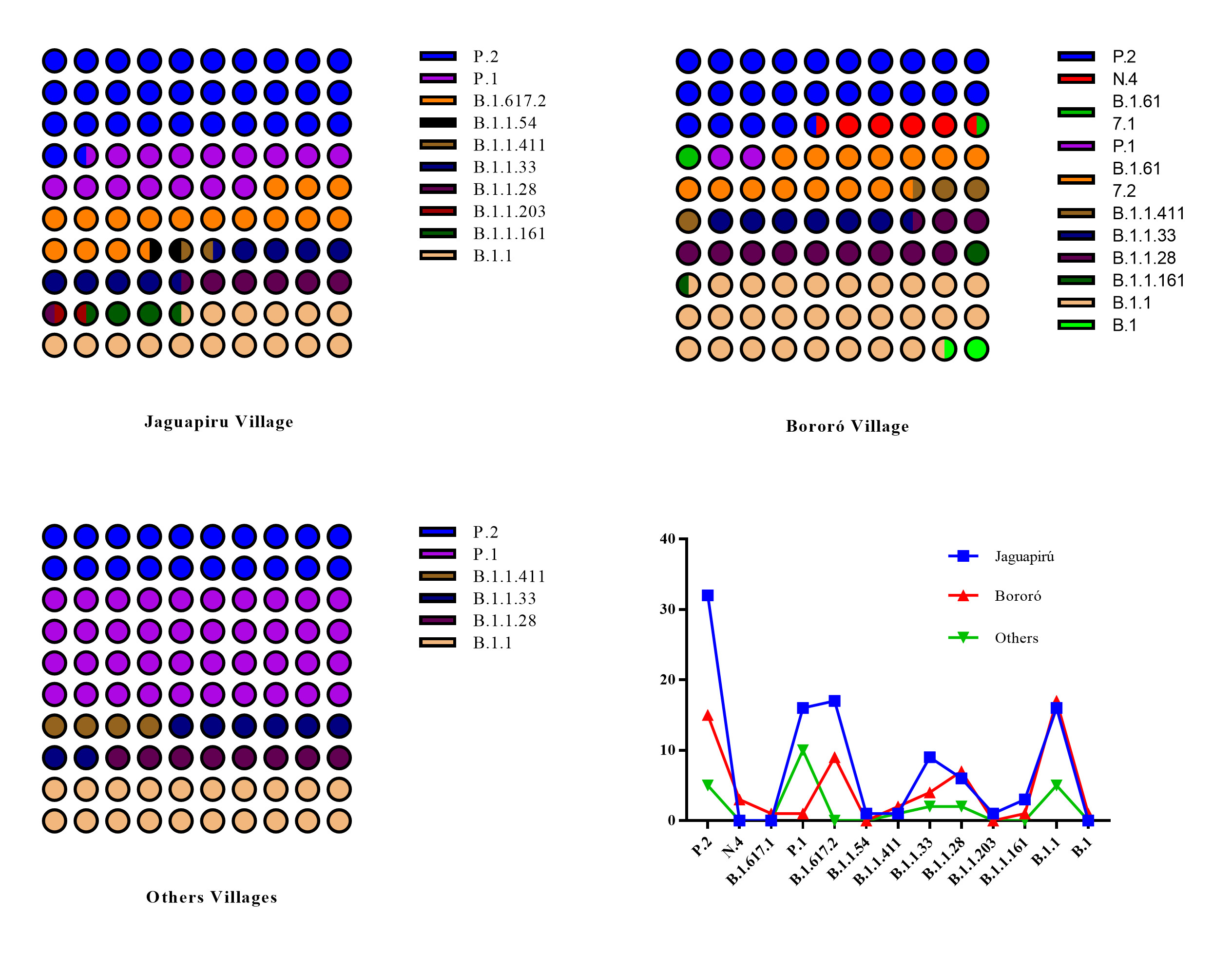

Supplement: Supplementary Figure 2 — Distribution of SARS-CoV-2 lineages related to the total number of positive individuals extracted by indigenous village in the municipality of Dourados - MS. [file Image_2.jpeg]
